# Illinois State Board of Health

**Published:** 1882-08

**Authors:** 


### ARTICLE VII.

ILLINOIS STATE BOARD OF HEALTH.

OFFICE OF THE SECRETARY, SHRINGFIELD, JULY 15, 1882.

DEAR SIR:—At a regular meeting of the State Board of Health, held April 13–15, 1882, the following resolution was adopted:

*Resolved*, That in order to protect the legal interests of survivors, to facilitate the detection of crime, and to secure fuller and more accurate knowledge of the causes of mortality, whereby preventive medicine and general sanitation may be promoted, the Illinois State Board of Health earnestly recommends to the proper authorities of all cities and towns in this State, having populations of one thousand or over, the enactment and enforcement of a suitable ordinance requiring a burial permit from a designated official, and based upon the physician's certificate of death, *now required by the statute*, as condition precedent to an interment within, or removal of a decedent without, the corporate limits of any such city or town.

A form of such ordinance is herewith presented, and it is hoped you may be able to secure its enactment.

It should be observed that wherever such an ordinance is adopted, the certifying physician is relieved of the necessity of transmitting his certificates direct to the county clerk, but will simply return them to the designated city or town official, who will forward them to the county clerk after using them as the basis for the burial permit. This has been found to work well practically in places where burial permits are required. It helps to secure a more general compliance with the law requiring physicians to report all deaths occurring under their supervision, with certificates of the causes thereof.

The manifest object of the State law is to secure such knowledge of the causes of mortality as may lead to measures for removing or modifying such causes as are susceptible of removal or modification. This is of primary importance to cities and towns, since a reputation for healthfulness or the reverse undoubtedly influences the growth and prosperity of any given locality. By means of the burial permit and its record, the facts contained in the physician's certificate may be made immediately available for this purpose, while they cannot be where returned direct to the county clerk. From the "suitable book" prescribed in section 5 of the ordinance, a weekly or monthly report may be compiled for publication, either in the newspaper press or otherwise, and thus the condition of, and the influences affecting, the public health may be accurately judged at any given time, and comparison made with other localities.

Where burial permits are required—as they are in many places—the existence of a contagious disease—as small-pox, scarlet fever, diphtheria—has often first been made known by the information given in the permit, which thus serves to direct preventive measures for arresting further spread of the contagion.

On the other hand, in the absence of burial permits many evils arise, among which may be mentioned the fact that the bodies of murdered persons may be more easily disposed of. Within a very brief period three such instances have come to the Secretary's knowledge, where the bodies of the victims were buried without exciting suspicion. Accidental clues led to disinterment and discovery of the crimes.

Briefly, the reasons for the enactment of such an ordinance may be thus summarized:

*First.*—It will be of value in securing fuller, more accurate, and more readily available knowledge of the causes of death—a knowledge which is absolutely necessary to the profitable application of efforts for the preservation of health, the limitation of disease and the prolongation of human life.

*Second.*—It will be of value in the protection of life against criminal violence, by facilitating the detection of such violence through preventing the burial of victims of homicide, abortion, poisoning, etc., without proper investigation.

*Third.*—It will be of value in the protection of property interests, by making the facts pertaining to a death and burial matters of record which may be useful in probating wills, settling estates, determining heirships, perfecting letters, adjusting life insurance, and kindred matters.

For the foregoing reasons, your interest and influence in behalf of the measure are confidently anticipated.

Very respectfully,

JOHN H. RAUCH, M.D., *Secretary.*

AN ORDINANCE IN RELATION TO BURIAL PERMITS.

Be it ordained by the —— of the —— of, ——, in the county of ——, in the State of Illinois:

1. That no burial or interment shall be lawful in the —— of, nor shall any dead body be removed from said ——, until a permit for such burial, interment or removal shall have been first obtained from the —— of said ——.

2. That such permit shall be issued by the —— upon his receipt of the usual certificate of death, signed by (1) the attending physician in the case; or, if none, by (2) one of the parents of the deceased; or, if none, by (3) the nearest of kin not a minor; or, if none, by (4) the resident householder where the death occurred; or, if none, by (5) any reputable citizen cognizant of the facts and circumstances of the death; or, if the death be the subject of an inquest, by (6) the coroner or other officer holding said inquest.

3. That any undertaker or sexton, and each and every other person engaged or concerned in a burial in violation of the provisions of this ordinance, and the officers and employés of any transportation company, or any other person or persons engaged or concerned in the removal of a dead body from said —— in violation of the provisions of this ordinance, shall be subject to a fine of not less than —— (—) dollars, nor more than ——(—) dollars for each offense.

4. That the —— shall enter in a suitable book, to be kept for that purpose, a record of all burial permits issued, specifying the date of issue and to whom issued, together with all the items of information contained in the certificates upon which the issue of such permits is based; and he shall forward to the county clerk of —— county, at the end of each month, all of said certificates so received during the month.

5. That this ordinance shall be in force from and after its passage and approval.

